# Utilization Patterns and User Characteristics of an Ad Libitum Internet Weight Loss Program

**DOI:** 10.2196/jmir.1347

**Published:** 2010-03-29

**Authors:** Martin Binks, Trevor van Mierlo

**Affiliations:** ^4^Dalla Lana School of Public HealthUniversity of TorontoTorontoONCanada; ^3^Evolution Health Systems IncTorontoONCanada; ^2^Division of Medical PsychologyDepartment of Psychiatry and Behavioral SciencesDuke University Medical CenterDurhamNCUSA; ^1^Binks Behavioral HealthPLLCHillsboroughNCUSA

**Keywords:** Weight loss, obesity, Web-based, self-help, Internet, utilization, intervention

## Abstract

**Background:**

The Internet holds promise for the delivery of evidence-based weight loss treatment to underserved populations. However, most studies do not reflect the more naturalistic and common *ad libitum*, or freely at will, use of the Internet. Randomized clinical trials, for example, typically include at least some direct contact with participants and often have restrictive selection criteria. There is a paucity of research examining utilization patterns of online weight loss programs, particularly in the rapidly expanding direct-to-consumer arena.

**Objectives:**

To examine self-reported characteristics (age, body mass index [BMI], gender), behaviors, and Internet site utilization patterns of a sample of users of a direct-to-consumer *ad libitum* Internet weight loss program.

**Methods:**

This study is based on analysis of archival data from the initial 15 weeks of an ongoing, free, evidence-based, direct-to-consumer Internet weight loss program, the Healthy Weight Center, which included standard information about nutrition, fitness, and behavioral strategies; monitoring tools; and moderated support group message boards. Participants encountered the program through self-directed Internet searches and anonymously registered to utilize the site. Self-reported user characteristics and electronically tracked utilization data were extracted from existing program data, compiled, and examined. Pearson correlations were computed to examine the association of program utilization with age and BMI. One-way analysis of variance (ANOVA) was used for gender comparisons.

**Results:**

We examined data from the first 204 adult users of the program who were classified as either overweight (BMI 25 to < 30 kg/m^2^) or obese (BMI ≥ 30 kg/m^2^). The mean age of participants was 42.0 years (SD 11.7), 81.9% (167/204) were women, and mean BMI was 32.01 kg/m^2^ (SD 6.26). The percent of participants who used program tools was as follows: 13.7%, meal planner; 10.8%, nutrition lookup: 17.6%, activity log; 14.2%, journal; and 22.1%, weight tracker. Participants also used the following educational resources: nutrition, 13.2%; fitness, 6.4%; and behavioral, 7.4%. Of the personal self-assessments available through the program, 57.8% of participants assessed personal barriers, and 50.5% assessed relationship with food. Only 7.8% used the support group message boards. No significant associations between site utilization and age, gender, or BMI were found. Reasons for wanting to lose weight were: health, 87%; appearance, 74%; mobility, 44%; doctor recommendation, 23%; and spouse/friend suggested, 12%. The age participants reported first becoming overweight was young adulthood, 31%; late adulthood, 28%; childhood, 22%; adolescence, 17%; and as a toddler, 3%. Self-perceived factors contributing to weight gain were lack of exercise for 70% of participants, emotions for 62%, overeating for 61%, and slow metabolism for 33%.

**Conclusions:**

Internet weight loss programs reach many people who cannot access traditional treatment. However, users appear not to be optimally utilizing key aspects of the weight loss intervention, such as education, monitoring, and support. This study provides insight into the patterns of *ad libitum* use of an online weight loss program across multiple treatment-related domains in a naturalistic Internet environment.

## Introduction

The Internet holds promise for the delivery of weight loss programs to underserved populations. However, the majority of published Internet weight loss research is from controlled clinical trials [1]. Clinical trial-based eHealth interventions may face challenges to generalizability relating to the inclusion of direct personal contact and extensive screening that is not part of a more naturalistic *ad libitum,* or freely at will, use of the Internet, as is the case with most Internet users’ experiences [2,3]. It also has been shown that eHealth programs are susceptible to poor utilization and attrition, which further limits their utility [4].

Internet-based weight loss research typically focuses on treatment outcomes, overall attrition rates, site logins and nutrition/weight tracking without consideration of the degree to which the therapeutic intervention (eg, accessing assessments, educational content, and support groups) was delivered [5]. If we are to develop effective Internet weight loss programs, we must ensure that the core treatment elements are being delivered. Individual tailoring of content through user self-assessments may be an effective approach to improving utilization. By using brief online questionnaires to assess the range of issues that may impact weight loss we may be able to deliver more personally relevant content to each individual user that addresses their particular area(s) of concern. Scientifically rigorous (ie, meta-analytic) review of research evaluating the efficacy of this approach is unavailable; however, narrative reviews have suggested that self-assessments may be too time consuming and thus unacceptable to the user [6,7]. Therefore, consideration of the degree to which participants use interactive self-assessments represents an important step in determining their feasibility.

In this analysis of archival data we examine website utilization data gathered from a free, evidence-based, anonymously utilized, direct-to-consumer weight loss program that includes nutrition, fitness, and behavioral information; self-assessments; monitoring tools; and moderated support group message boards.

## Methods

### Setting and Program Description

The Healthy Weight Center [8] is a free access, evidence-based [9,10] Internet weight loss program that provides nutrition, fitness, and behavioral information; monitoring tools; interactive assessments with individually tailored feedback; and moderated support group message boards (see Multimedia Appendix 1). The Internet intervention is based on a review of design principles and consumer testing across multiple eHealth platforms (ie, smoking cessation, depression, panic disorder, and problem drinking) and usability testing of this and our other eHealth platforms [11]. The program utilizes free-form matrix design, that is, all program elements are available to every user. The program also provides an online program guide to facilitate full utilization of the program elements.

The program was available for free to anyone having access to the World Wide Web from any geographic region of the world. There was no advertising or promotion of the program. A link to the program was added to the Evolution Health corporate site [12], and potential users could also encounter the program using publicly available Internet searches. The period of data collection for this study was the initial 15-week period of program availability from May to Sept 2008. The program continues to be available to the public. There was no formal recruitment for the program. To enroll, users completed the online registration process consisting of 14 questions that included weight, height, date of birth, gender, email address, occupation and questions relating to weight and dieting history. Registrants were also asked to electronically endorse the program disclosure agreement explaining that data were being collected anonymously and that this unidentifiable information would be used for research purposes.

### Subjects

The subjects of this study were the first 204 individuals who registered for the online weight loss program and who met the following inclusion criteria: (1) 18 years or older, (2) body mass index (BMI) classified as overweight (BMI 25 to < 30 kg/m^2^) or obese (BMI ≥ 30 kg/m^2^); (3) completion of all questions contained in the online registration process; and (4) endorsement of the program disclosure agreement.

Of these 204 participants, 18.1% (37/204) were men, and 81.9% (167/204) were women. The mean age of the participants was 42.0 (SD 11.7) years; 44.4 (SD 12.6) years for men, and 41.5 (SD 11.5) years for women. The mean BMI of the participants was 32.0 (SD 6.3) kg/m^2^; 31.2 (SD 5.2) kg/m^2^ for men, and 32.2 (SD 6.5) kg/m^2^ for women.

Mean weight of the participants was 89.4 (SD 19.9) kg; 99.8 (SD 16.8) kg for men, and 86.9 (SD 19.3) kg for women. All registrants with complete data were included for analysis (N=204).Thirty-three subjects were excluded for missing (BMI) data.

### Data Collection

Data for the 204 registrants meeting the inclusion criteria were extracted from the existing Healthy Weight Center database. All online registration questionnaires for the Healthy Weight Center program adhered to international privacy guidelines [13,14]. Procedures were in accordance with the Helsinki Declaration of 1975, as revised in 2008 [15]. Because the study was based on the use of unidentifiable archival data, the study was determined to be exempt from further review.

Personal characteristics and behaviors were based on registrants’ self-reported responses to the online questionnaires. We used website analysis tools to determine the extent of program utilization for each participant. Utilization of each individual program element was defined as accessing that program element at least once: educational information (nutrition, fitness, and behavioral), self-assessments (personal barriers and relationship with food) [16], monitoring tools (weight tracker, exercise tracker, journal, and nutrition lookup), and the moderated support groups.

### Statistical Analysis

Personal characteristics/behaviors and utilization data were tabulated as percent reporting. To explore the influence on site utilization of age (eg, younger individuals may be more Internet savvy and thus engage in higher utilization) and BMI (eg, heavier individuals may be more motivated and thus engage in higher utilization), Pearson correlations were computed. One-way analysis of variance (ANOVA) was used to consider gender differences in demographic factors (ie, age, BMI) and program utilization.

## Results

### Gender

One-way ANOVA revealed no significant differences by gender for age (*F*_1,202_ = 1.8, *P* = .18), BMI (*F*_1,202_ = 0.7, *P* = .40), and program utilization (*F*_1,202_ = 0.4, *P* = .53).

### Program Utilization, Age, and BMI

The mean number of program elements utilized was 2.7 (SD 3.9). Pearson correlations revealed no significant correlations of program utilization with age (N = 204; *r* = -.02; *P* = .78) or BMI (N = 204; *r* = -.02; *P* = .81).

### Utilization Patterns

Site utilization data for all users (N= 204) based on accessing each tool at least one time is presented in Table 1. Site tools used for monitoring key weight loss behaviors (meal planner, nutrition lookup, activity log, journal and weight tracker) were not highly utilized. Similarly, educational materials (nutrition, fitness, and behavioral) and support group message boards were not accessed by the majority of users. However, interactive assessments (personal barriers and relationship with food), showed relatively higher utilization.

### Personal Characteristics/Behaviors

Data concerning personal characteristics/behaviors were compiled for all participants (N = 204). When participants were asked to indicate all applicable reasons for wanting to lose weight 87% (177/204) endorsed health; 74% (151/204), appearance; 44% (89/204), mobility; 23% (47/204), doctor recommended; and 12% (24/204) indicated that a friend/spouse suggested they lose weight. In terms of the age that users reported first becoming overweight, 31% (63/204) reported young adult onset, 28% (57/204) reported late adult onset, 22% (44/204) reported onset during childhood, 17% (34/204) reported adolescent onset, and 3% (6/204) reported first becoming overweight as a toddler. Of the factors that participants believed contributed most to their weight gain, the most frequently endorsed items were: lack of exercise, endorsed by 70% (143/204); emotions, endorsed by 62% (126/204); overeating, endorsed by 61% (124/204); and slow metabolism, endorsed by 33% (67/204).

**Table 1:** Utilization of program elements (N=204)

Program Element (see Multimedia Appendix 1)	Utilization
	n (%)
Meal planner	28 (13.7)
Nutritional data look up tool	22 (10.8)
Activity log	36 (17.6)
Journal	29 (14.2)
Weight tracker	45 (22.1)
Nutrition education	27 (13.2)
Fitness education	13 (6.4)
Behavioral education	15 (7.4)
Support group message board	16 (7.8)
Personal barriers assessment	118 (57.8)
Relationship with food assessment	103 (50.5)

## Discussion

Internet weight loss programs that provide evidence-based intervention can reach many individuals who have limited access to traditional treatments. However, website users may not be utilizing Web-based weight loss resources optimally. The standard of care in weight management involves nutrition, fitness, and behavioral education; behavioral self-monitoring; and use of personal support [9,10]. In our study of *ad libitum* use of a Web-based weight loss program, participants did not take full advantage of these essential treatment elements.

In our Internet weight loss program, interactive self-assessments (eg, personal barriers and relationship with food) were more highly utilized than the similarly interactive weight/fitness tracking tools and journaling. This finding suggests that interactivity alone was not likely responsible for higher utilization. A distinguishing feature of our interactive self-assessments was the promise of personally relevant feedback, which may have made these exercises particularly appealing to the user. Evidence from previous studies suggests that tailoring interventions to individuals may be an effective tool in improving health behavior, but concerns arise about the acceptability to participants of completing the self-assessments that are needed to tailor content [6,7,17]. Our findings suggest that users may actually prefer using interactive self-assessments as compared with other program elements that were available to them in our study (monitoring tools, educational content, support group message boards). This finding highlights the need for architects of Internet-based programs to explore increasing the use of interactive exercises to tailor the user experience in ways that increase personal relevance. This may be preferable to relying on predetermined blocks of educational content to deliver the necessary treatment elements. Furthermore, identifying relevant baseline personal characteristics/behaviors of users may also deserve further consideration. For example, in this study, the majority of users reported that health, mobility, and appearance were important contributors to their decision to engage in the program. Perhaps by tailoring interactive assessments and content to areas of relevance to the individual user, we may be able to approximate the more typical in person health care experience.

Finally, while we did not consider this directly in our study, the literature suggests that utilization of therapeutic program elements may be increased through novel approaches to enhancing the user experience and including incentives such as rewards for completing exercises. Architects of future Internet-based weight loss programs should consider these strategies [18,19,20].

### Strengths and Limitations

This study provides insight into how individuals utilize Internet resources in a natural setting as opposed to a clinical trial environment. The relatively fewer barriers to enrollment, based on this program’s open availability on the Internet in comparison with the barriers to entry that are typical of Internet-based clinical trials, is a particular strength. Also, few studies have examined the utilization of specific program elements (eg, educational materials, tools, and assessments), focusing instead on broader criteria such as the total number of log-ins. Limitations of our study include the small sample size in comparison with other studies of natural use of Internet health programs and lack of information about users’ geographic area (eg, country of residence) each of which may limit generalizability. Another limitation is the lack of information about treatment outcomes and our inability to determine the frequency (multiple accessing of each element) or extent (amount of time spent using each element) with which elements of the program were used, as our data was based on accessing each element at least once. These factors are important considerations for future studies.

### Summary

This study provides insight into potential strategies for optimizing program design to improve utilization of core treatment elements of Internet-based weight loss programs. This is a necessary step to ensure quality when widely disseminating weight loss treatments. Future studies should include detailed analysis of utilization patterns and their relationship to both short- and long-term outcomes.

## Multimedia Appendix 1

Screenshots from the Healthy Weight Center Internet site illustrating the program elements outlined in Table 1
