# Supplementary material for: Utilization Patterns and User Characteristics of an Ad Libitum Internet Weight Loss Program
Source: J Med Internet Res. 2010 Mar 29;12(1):e9. doi: 10.2196/jmir.1347 (PMC2872772; doi:10.2196/jmir.1347)
Supplement: Supplementary file 1 [file jmir_v12i1e9_app1.ppt]

## Slide 1
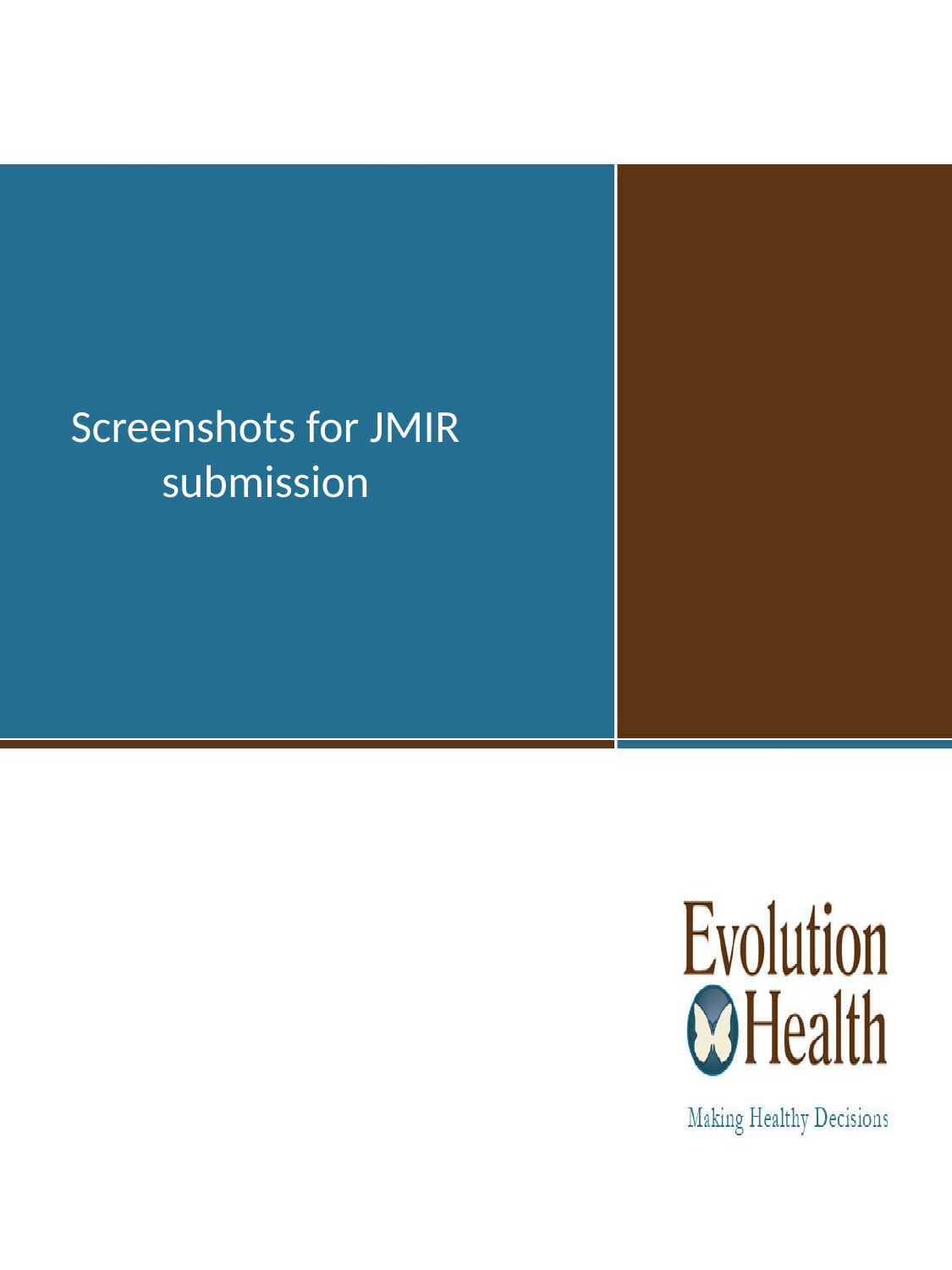

# Screenshots for JMIR submission

## Slide 2
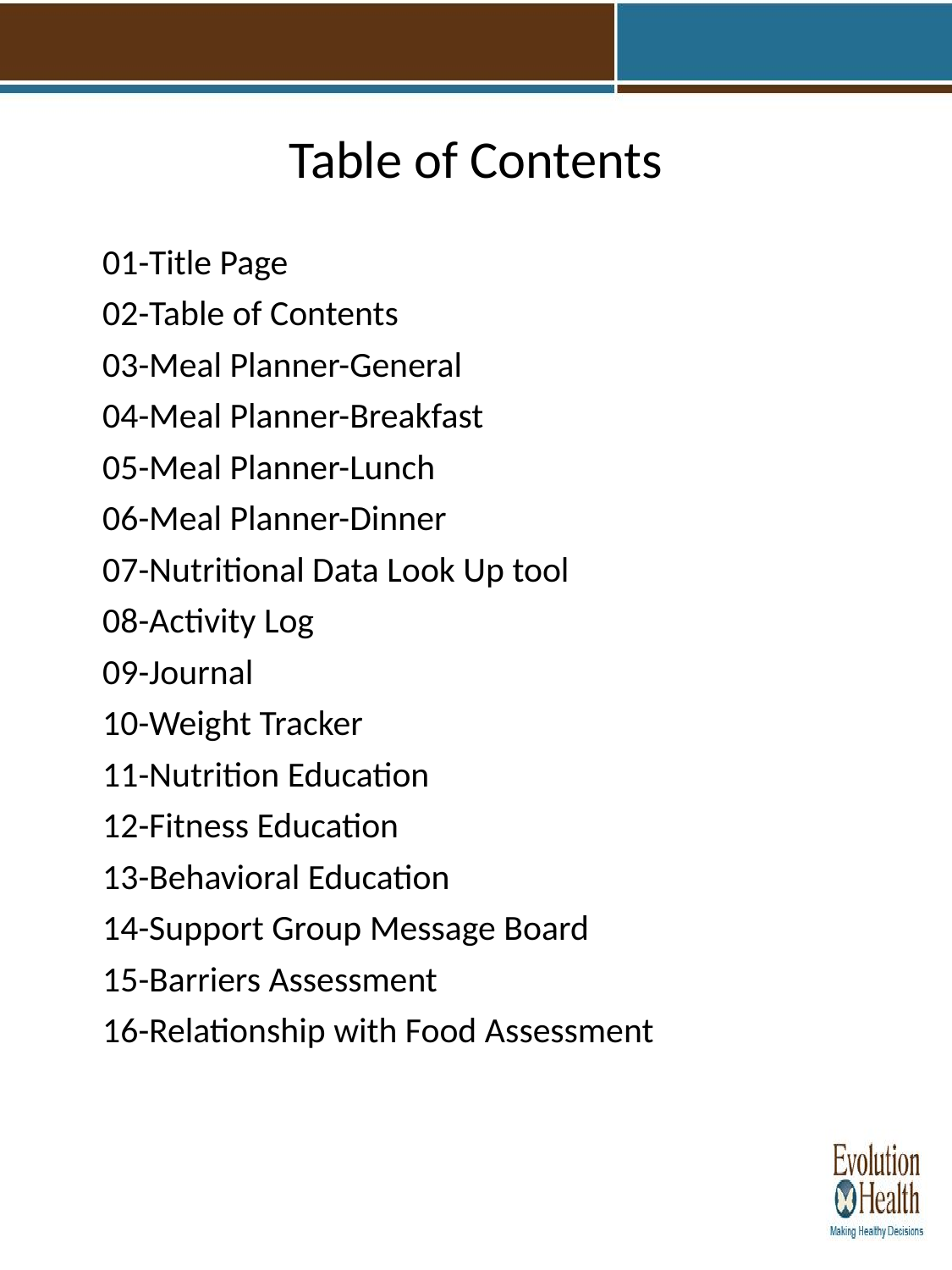

# Table of Contents
01-Title Page
02-Table of Contents
03-Meal Planner-General
04-Meal Planner-Breakfast
05-Meal Planner-Lunch
06-Meal Planner-Dinner
07-Nutritional Data Look Up tool
08-Activity Log
09-Journal
10-Weight Tracker
11-Nutrition Education
12-Fitness Education
13-Behavioral Education
14-Support Group Message Board
15-Barriers Assessment
16-Relationship with Food Assessment

## Slide 3
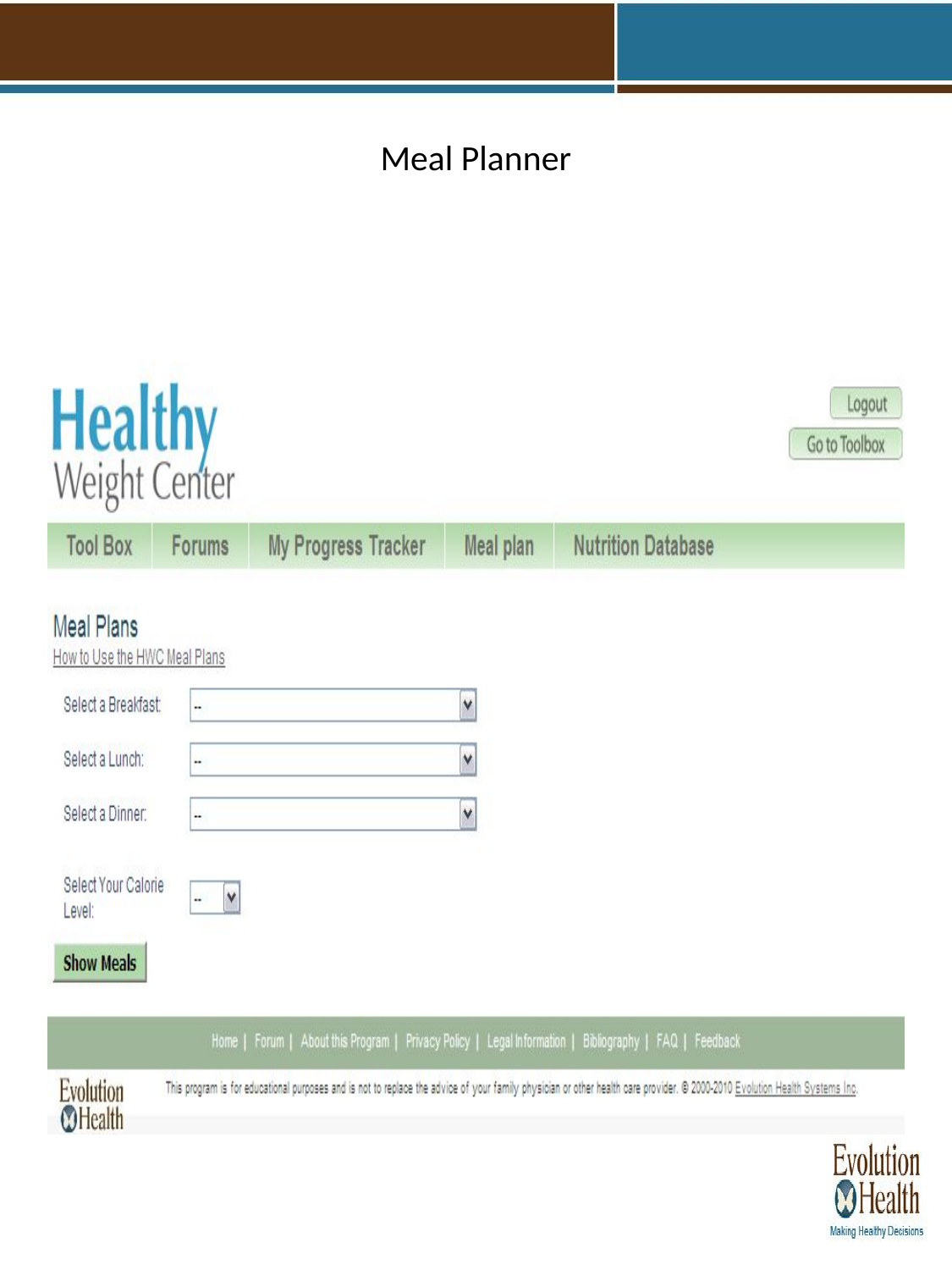

# Meal Planner

## Slide 4
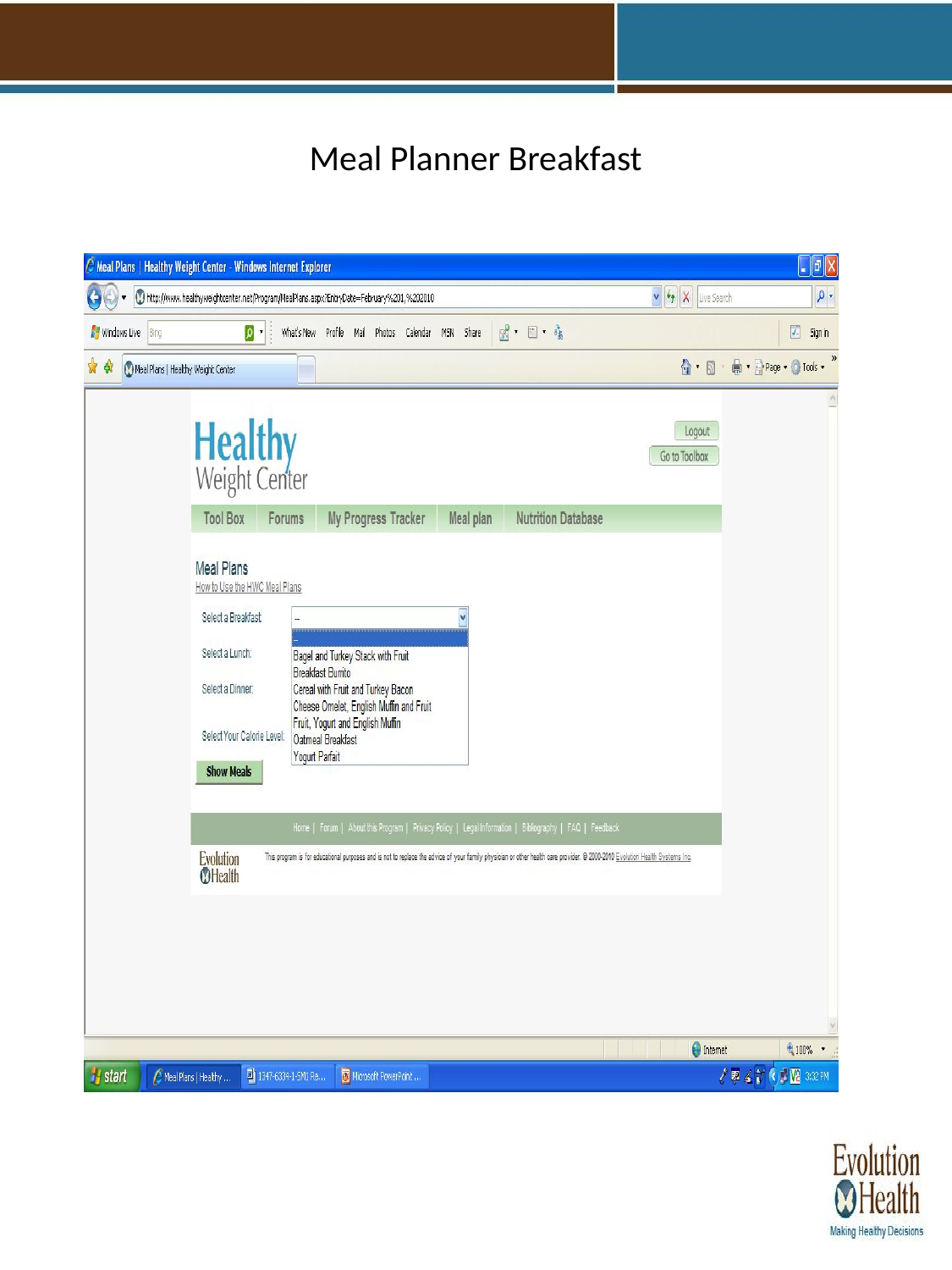

# Meal Planner Breakfast

## Slide 5
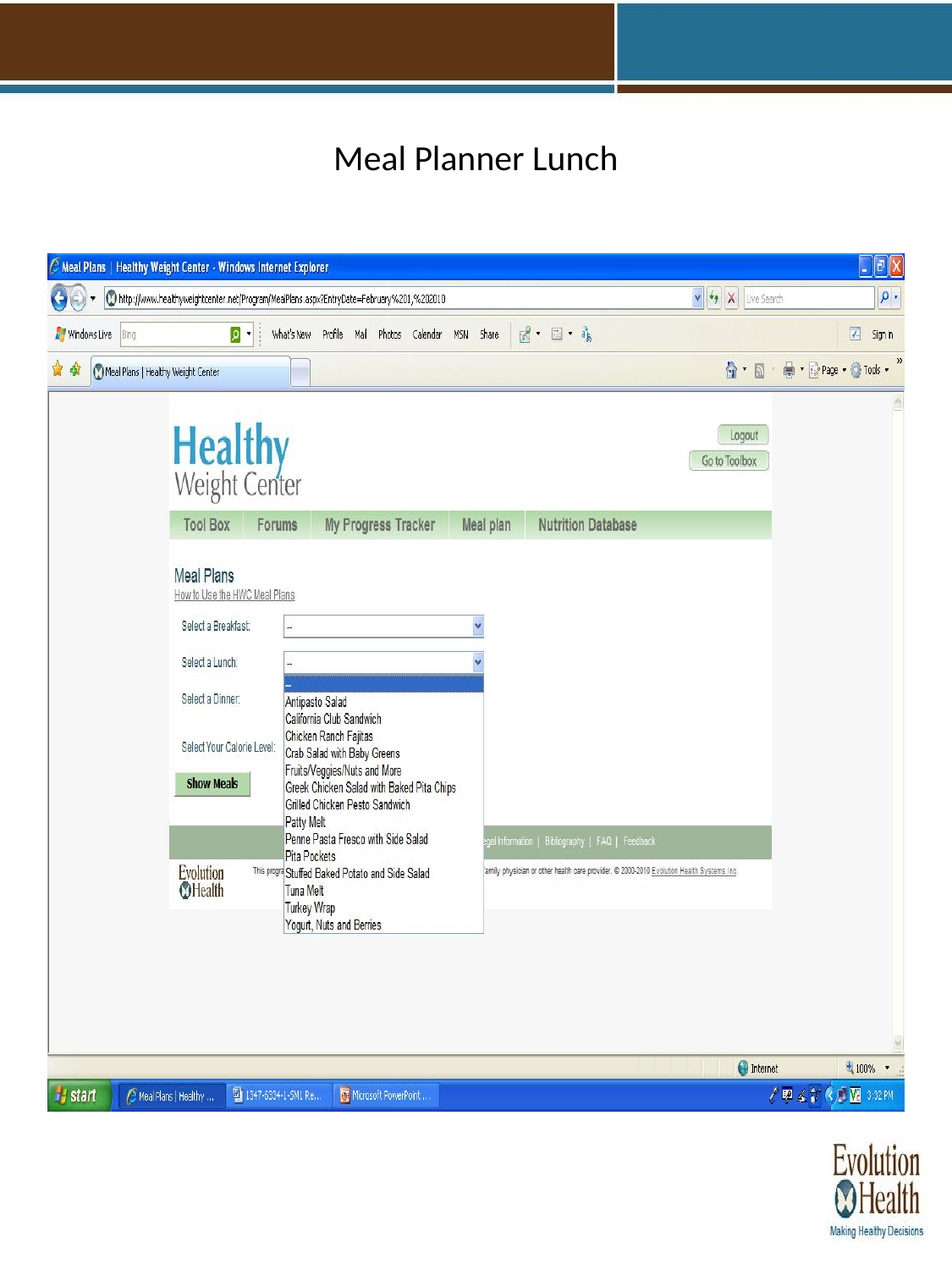

# Meal Planner Lunch

## Slide 6
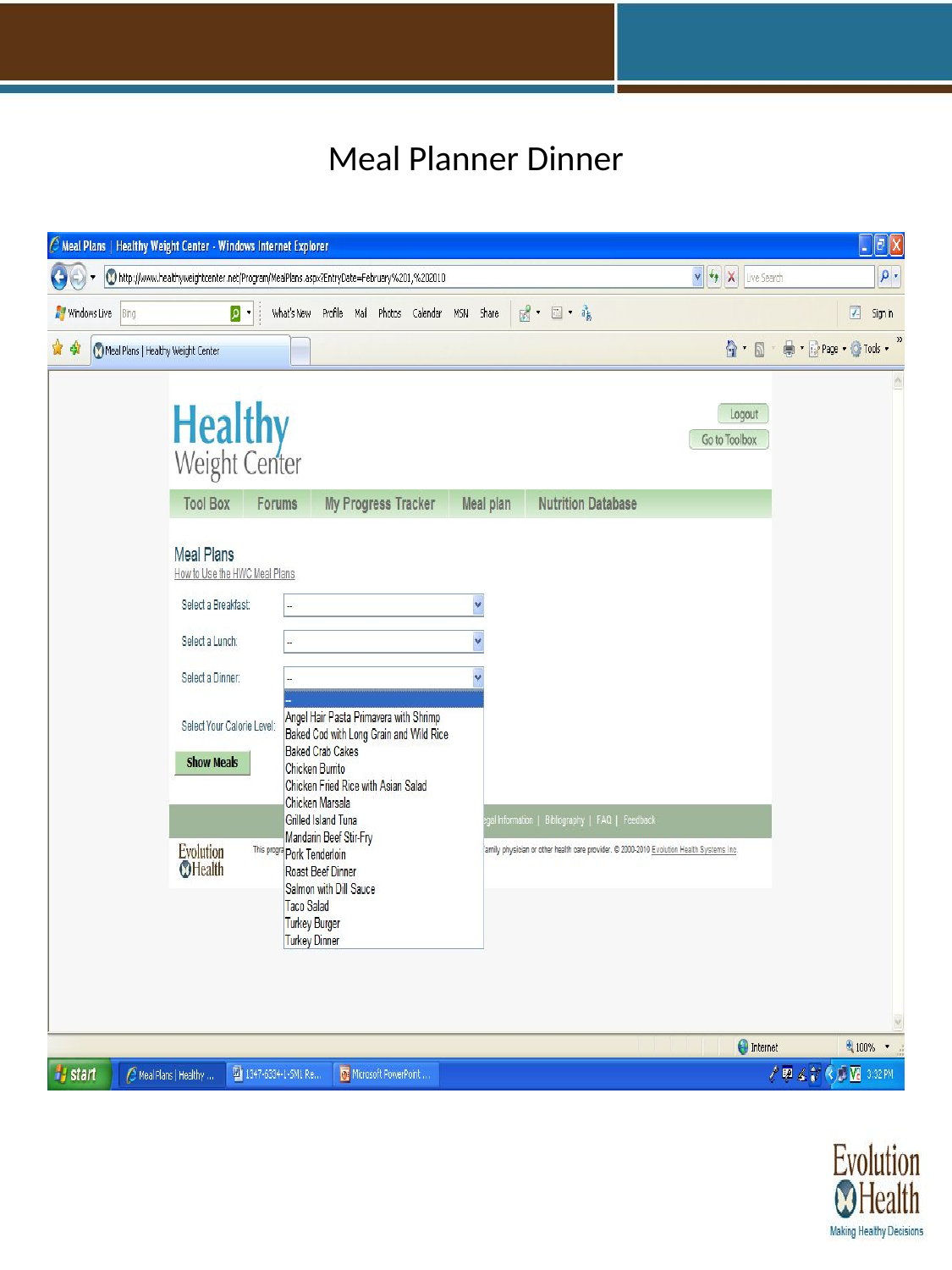

# Meal Planner Dinner

## Slide 7
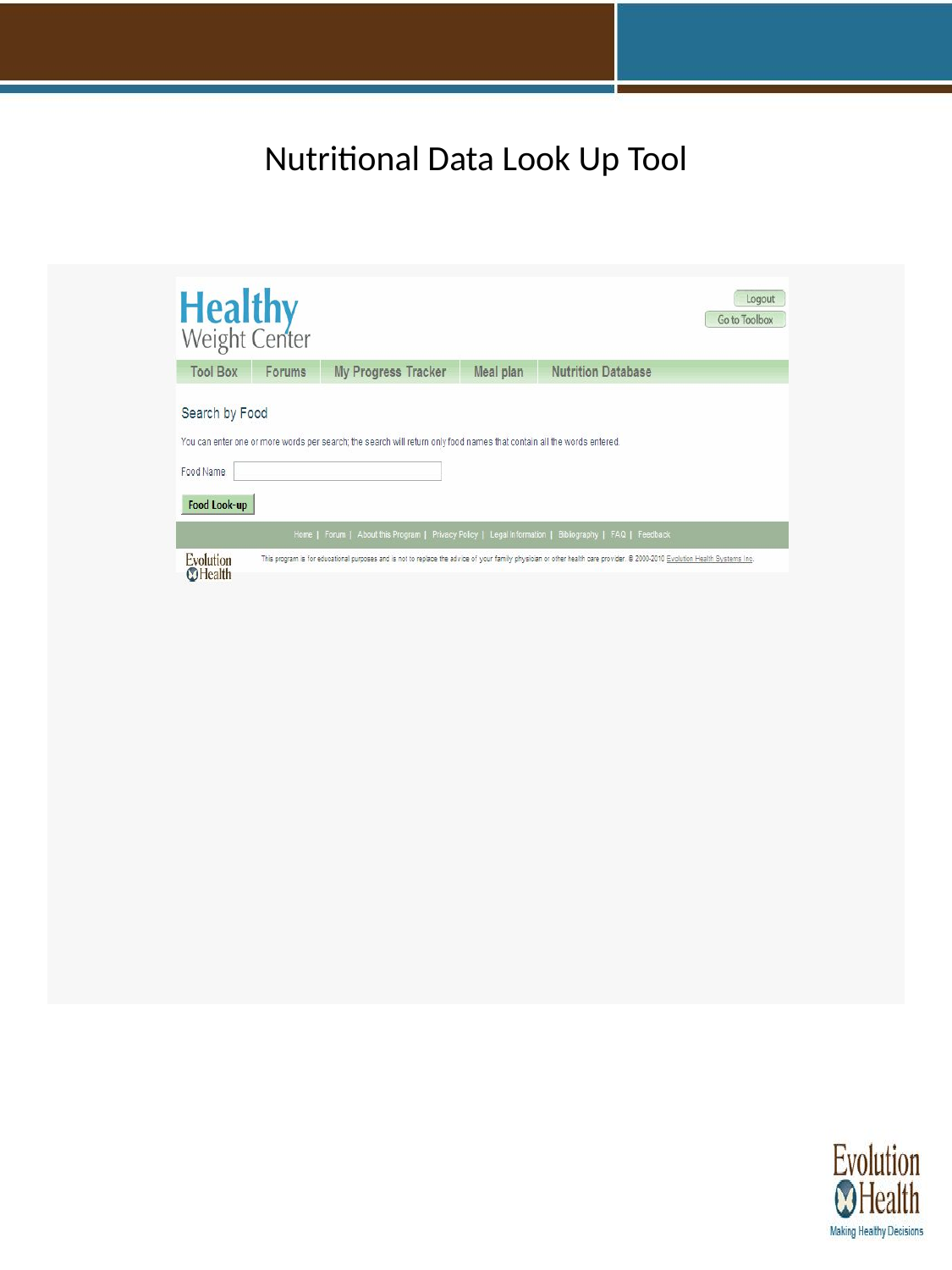

# Nutritional Data Look Up Tool

## Slide 8
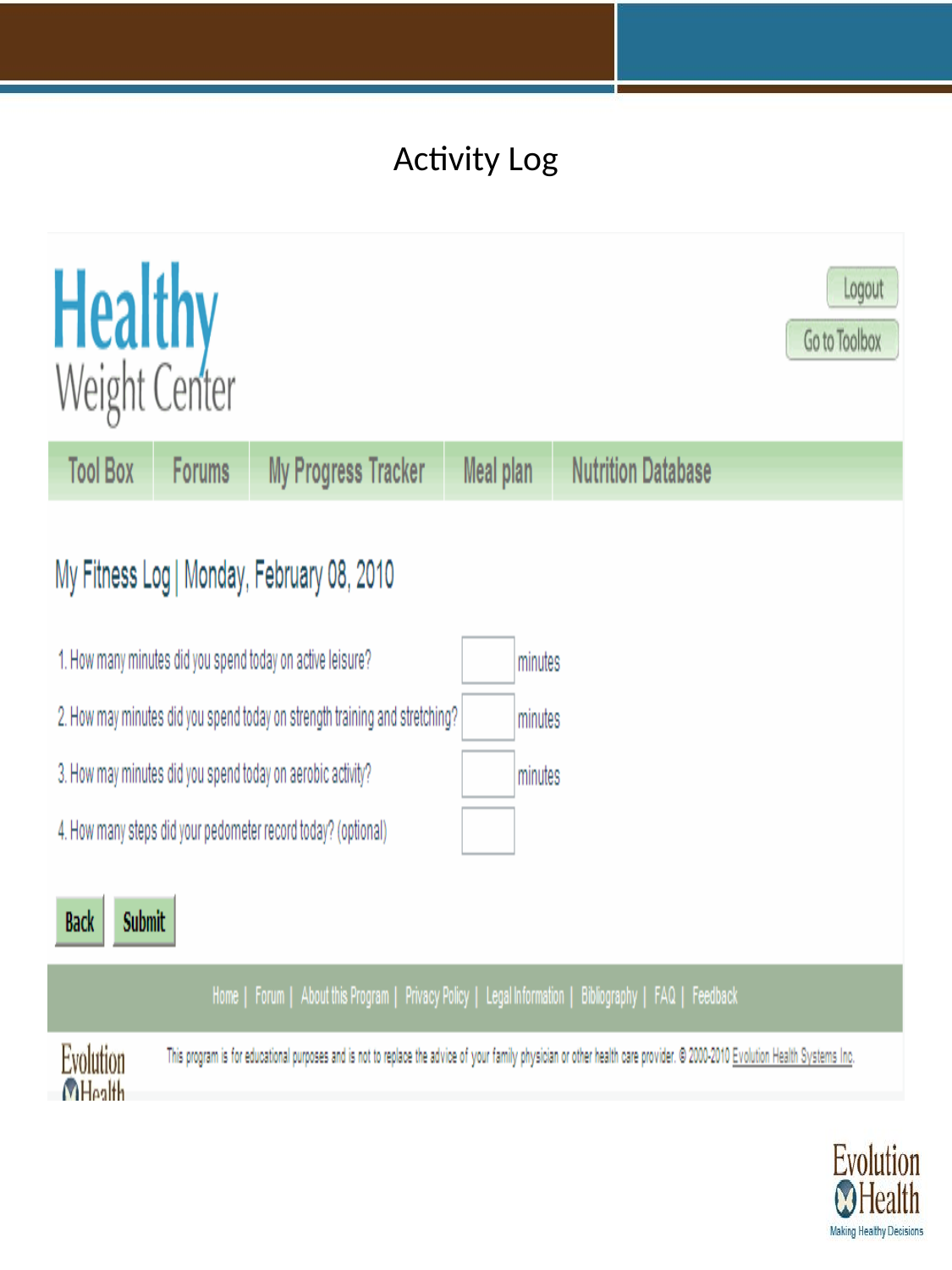

# Activity Log

## Slide 9
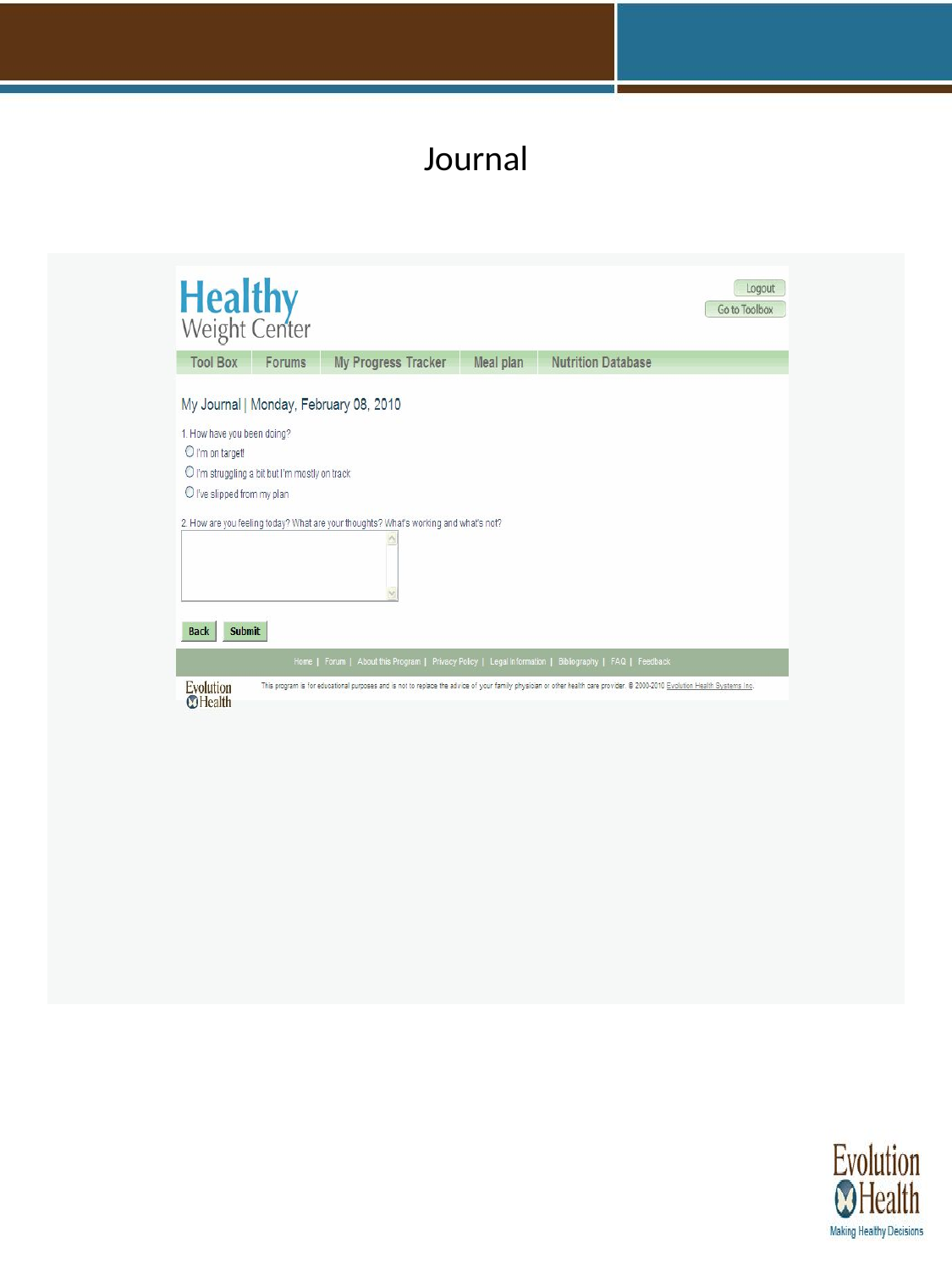

# Journal

## Slide 10
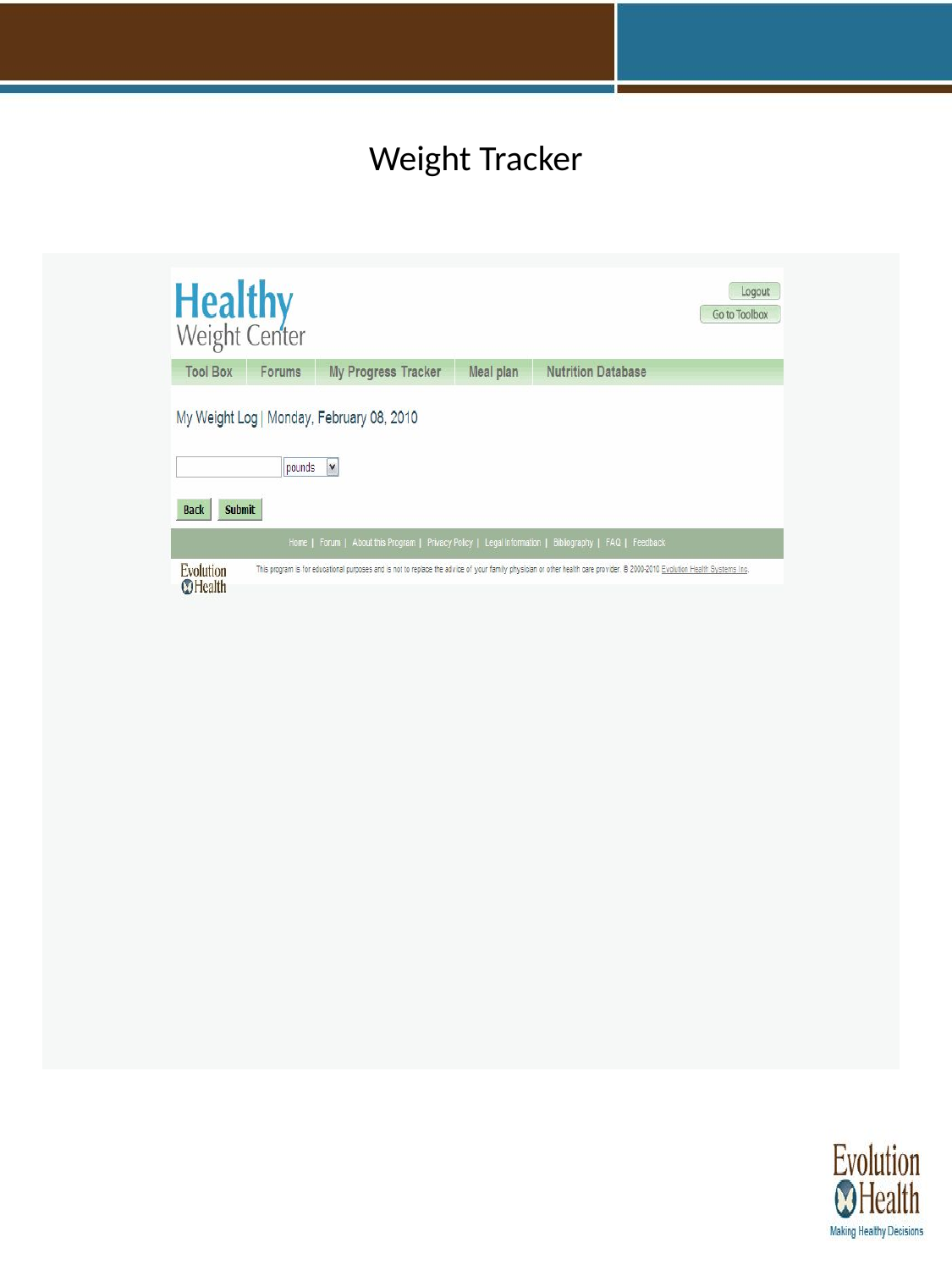

# Weight Tracker

## Slide 11
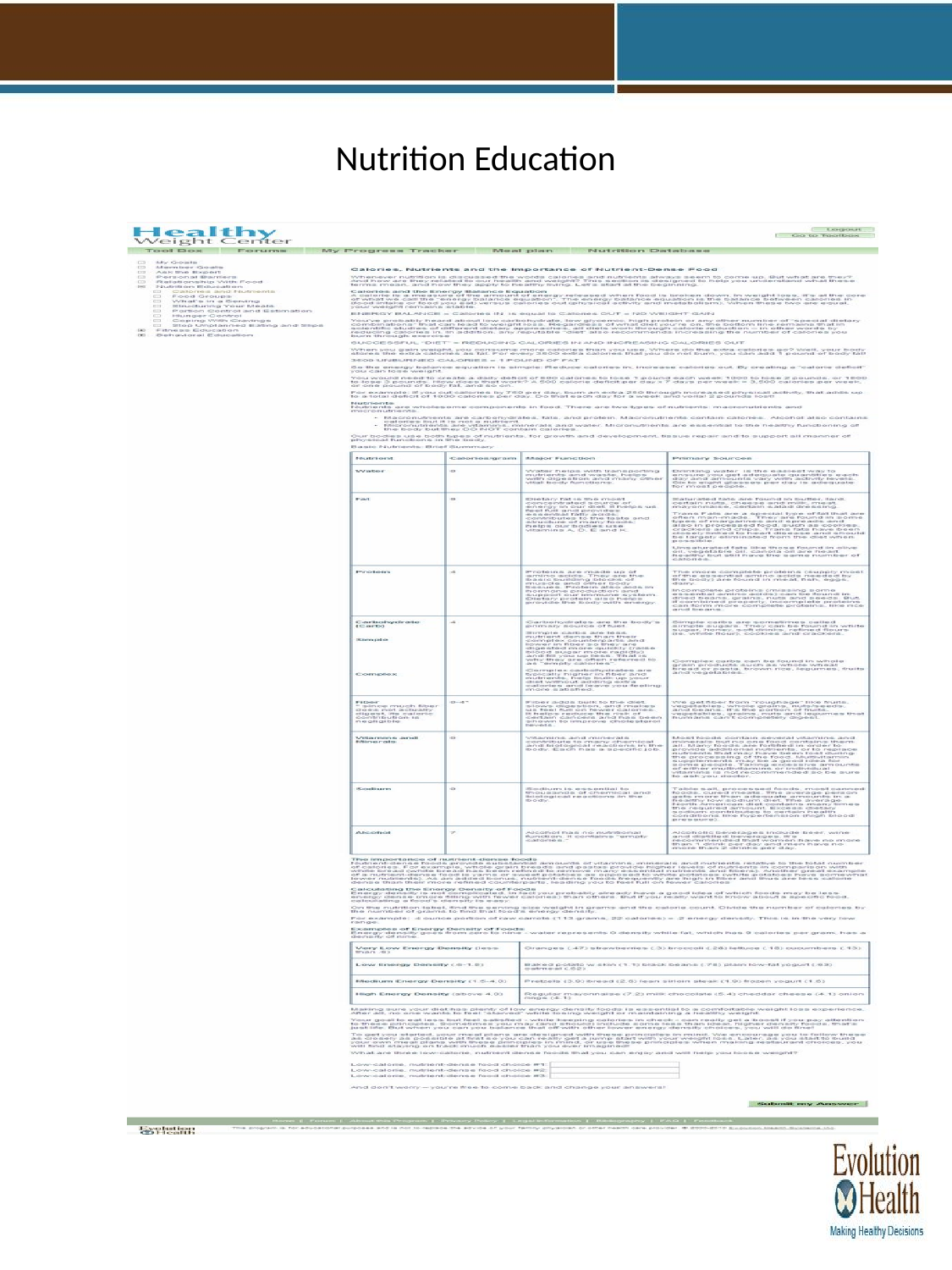

# Nutrition Education

## Slide 12
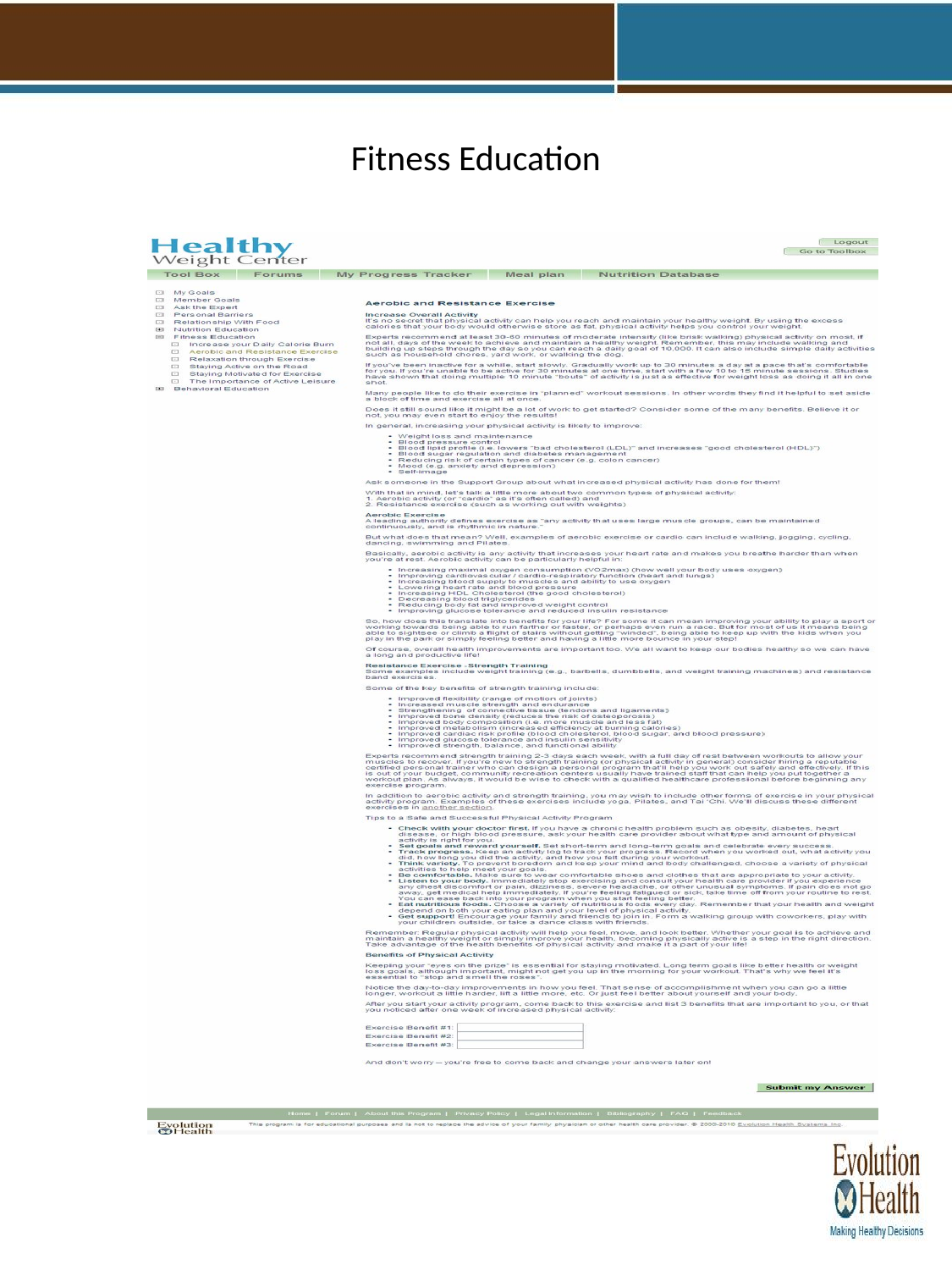

# Fitness Education

## Slide 13
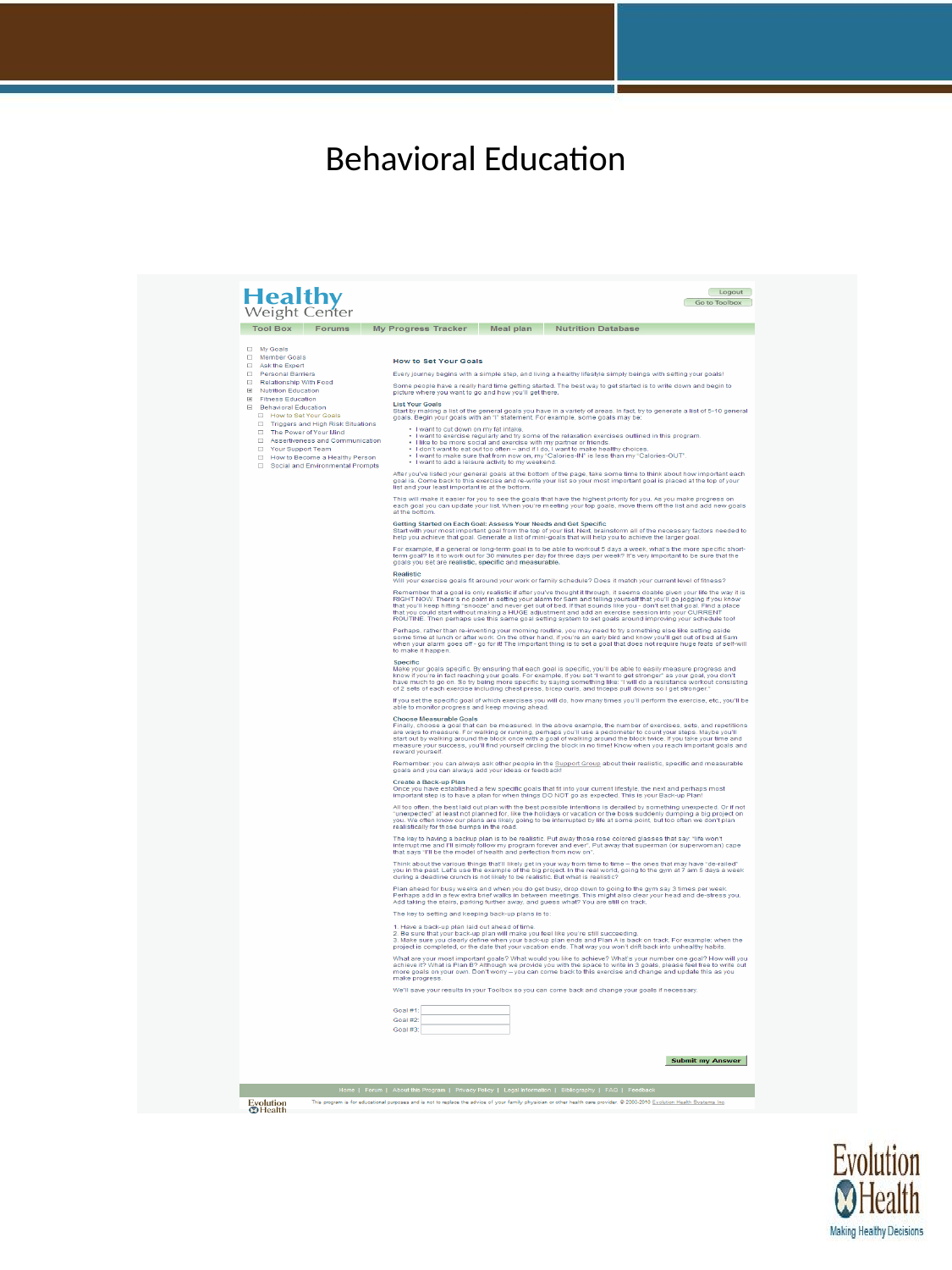

# Behavioral Education

## Slide 14
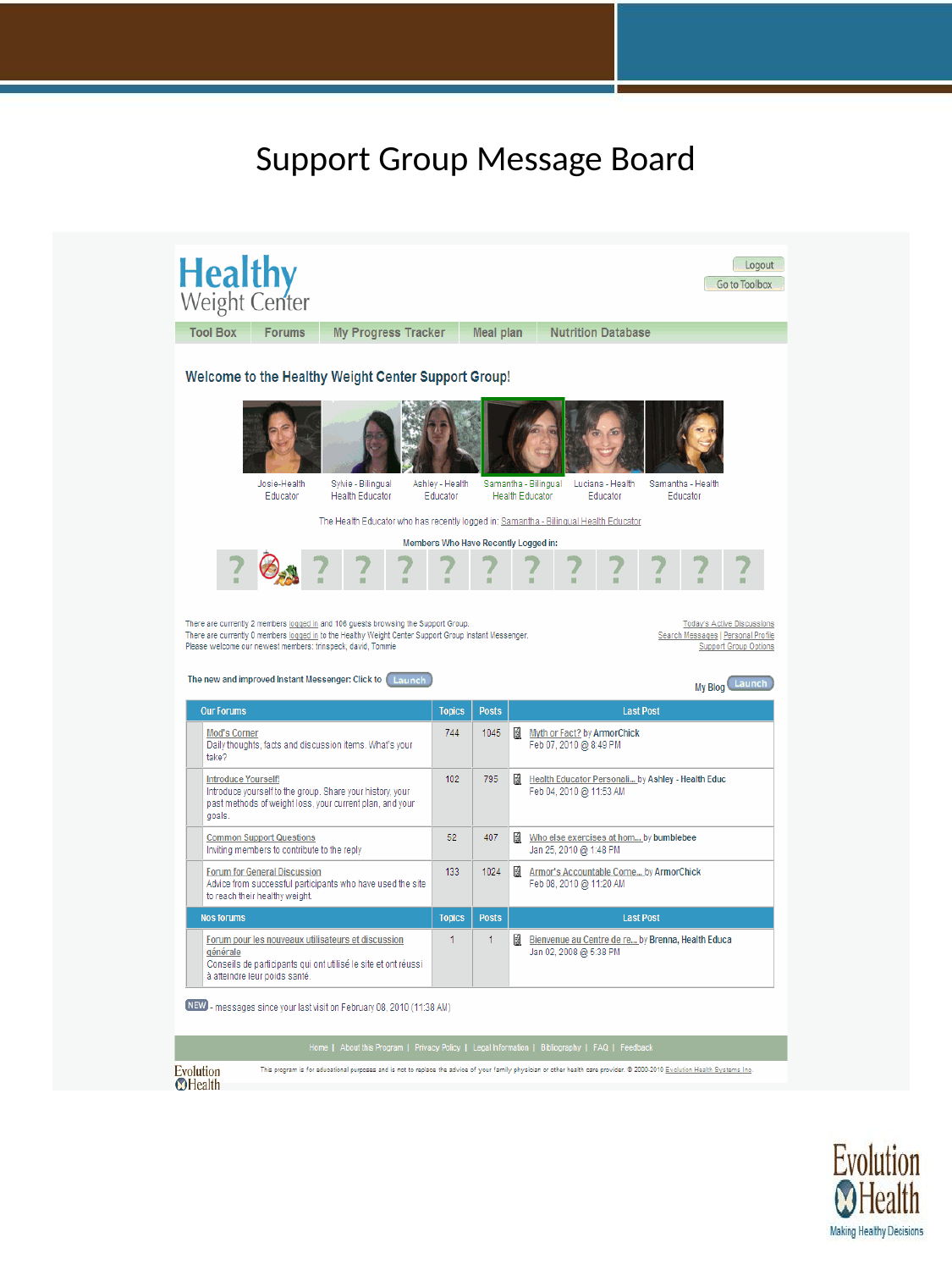

# Support Group Message Board

## Slide 15
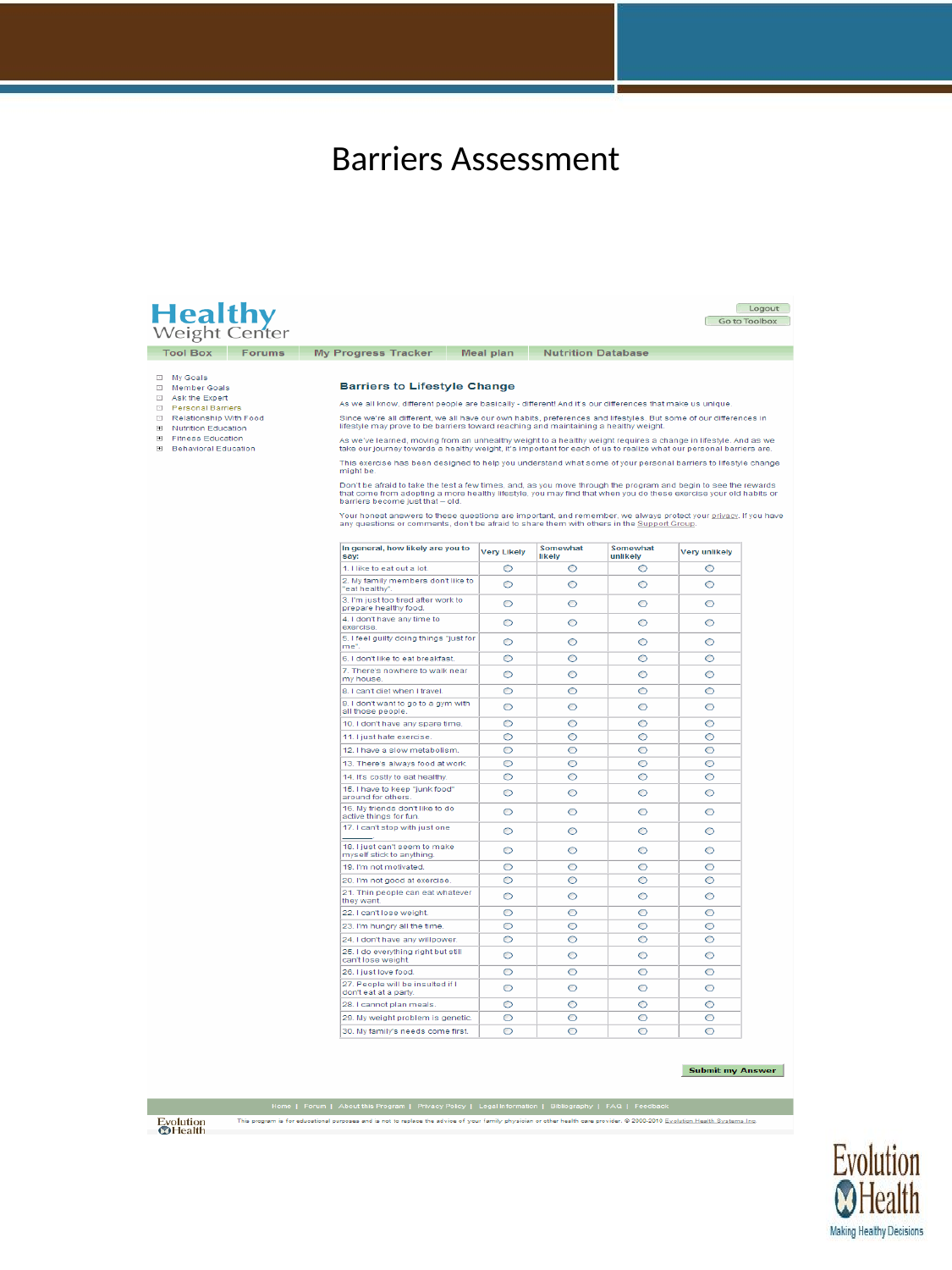

# Barriers Assessment

## Slide 16
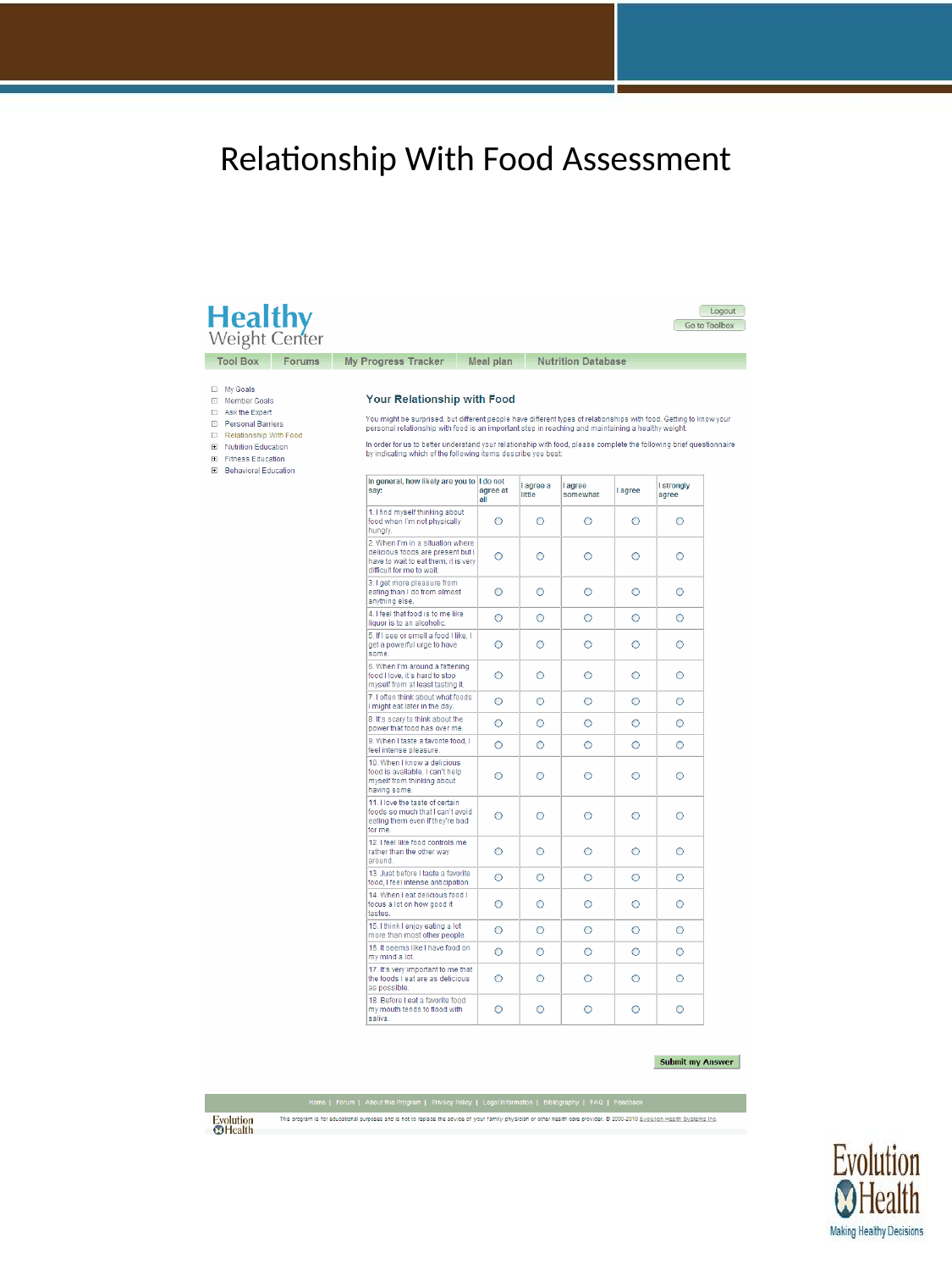

# Relationship With Food Assessment
